# Local data of heat flux, wall temperature and the void phase along the boiling curve during vertical subcooled flow boiling of refrigerant Novec 649 at a copper wall

**DOI:** 10.1016/j.dib.2018.10.138

**Published:** 2018-11-01

**Authors:** Moritz Bruder, Ludwig Sembach, Veronika Krumova, Thomas Sattelmayer

**Affiliations:** Lehrstuhl für Thermodynamik, Technische Universität München, Boltzmannstr. 15, 85748 Garching bei München, Germany

**Keywords:** CFD, Computational fluid dynamics, FDB, Fully developed boiling, FDFB, Fully developed film boiling, CHF, Critical heat flux, PIF, Phase indicator function

## Abstract

This data set contains local experimental data of heat flux, wall temperature and void data profiles for vertical subcooled flow boiling of refrigerant Novec 649 at a copper wall. This data article presents average boiling curves from single-phase convection to fully developed film boiling for six combinations of mass flux and subcooling. Void profiles are provided for void fraction, void detection frequency, void velocity and void ligament size for three characteristic states along each boiling curve. Thermocouples were used to measure heat flux and temperature. Optical single fiber and double fiber micro probes were used for obtaining void data profiles. A traversing mechanism was used to position the optical fiber micro probes relative to the heater surface.

## Nomenclature

**Greek letters**
$\Delta h_{l,g}$: Specific enthalpy of evaporation kJ/kg
Δ: Denotes a difference −
λ: Thermal conductivity $Wm^{-1}K^{-1}$
ρ: Density $\mathrm{kg}m^{-3}$
σ: Surface tension $\mathrm{mN}m^{-1}$
τ: Contact time s

**Roman letters**
$\dot{q}$: Surface heat flux $Wm^{-2}$
c: Specific heat capacity $\mathrm{J k}g^{-1}K^{-1}$
f: Frequency Hz
G: Mass flux $\mathrm{kg}m^{-2}s^{-1}$
i: Index variable −
j: Index variable −
k: Index variable −
l: Length m
N: Quantity −
n: Number of samples −
p: Pressure $Nm^{-2}$
sr: Sample rate Hz
T: Temperature K
t: Time s
U: Voltage V
u: Velocity $ms^{-1}$
y: Distance in wall normal direction m

**Subscripts**
crit: Conditions at critical point
cu: Copper
g: Gas phase
l: Liquid phase
lig: Void ligament
p: Isobaric
probe: Refers to a characteristic of a fiber optic probe
sat: Saturation conditions
sub: Refers to inlet subcooling
th: Threshold
w: Refers to the wall

**Specifications table**

**Table:** 

Subject area	*Two-phase flow, heat transfer*
More specific subject area	*Boiling phenomena, critical heat flux*
Type of data	*Figures and processed data as text files containing comma separated values.*
How data were acquired	*Thermocouples for heat flux and temperature data, fiber optical micro probes for local void data profiles.*
Data format	*Analyzed*
Experimental factors	*The void data profiles were obtained from raw optical fiber probe data, which are voltage signals between a minimum of 0 V and a maximum of 10 V. Prior to the acquisition of the raw data for each experiment, each individual fiber probe channel was set up to achieve an optimum signal to noise ratio.*
Experimental features	*Boiling curves and profiles of void data were obtained at different mass fluxes and subcoolings above a copper heater mounted flush in a vertical rectangular flow channel.*
Data source location	*Lehrstuhl für Thermodynamik, Technische Universität München, Boltzmannstr. 15, 85748 Garching bei München, Germany*
Data accessibility	*The data are presented within this article as figures and it is available as*Supplementary material*in the online version of this article.*
Related research article	*None*

**Value of the data**

• These data consist of average boiling curves and detailed quantitative local information about the void phase in the cross section of a flow channel for refrigerant Novec 649.
• The presented void profiles for void fraction, void detection frequency, void velocity and void ligament size can provide insights into the critical heat flux process in subcooled flow boiling.
• Average boiling curves and void profiles are valuable for the development and validation of new predictive numerical tools for the simulation of two-phase flow.
• With this data important CFD-validation data such as interfacial area density or sauter diameters can be derived.

### 1 Data

**Fig. 1:**
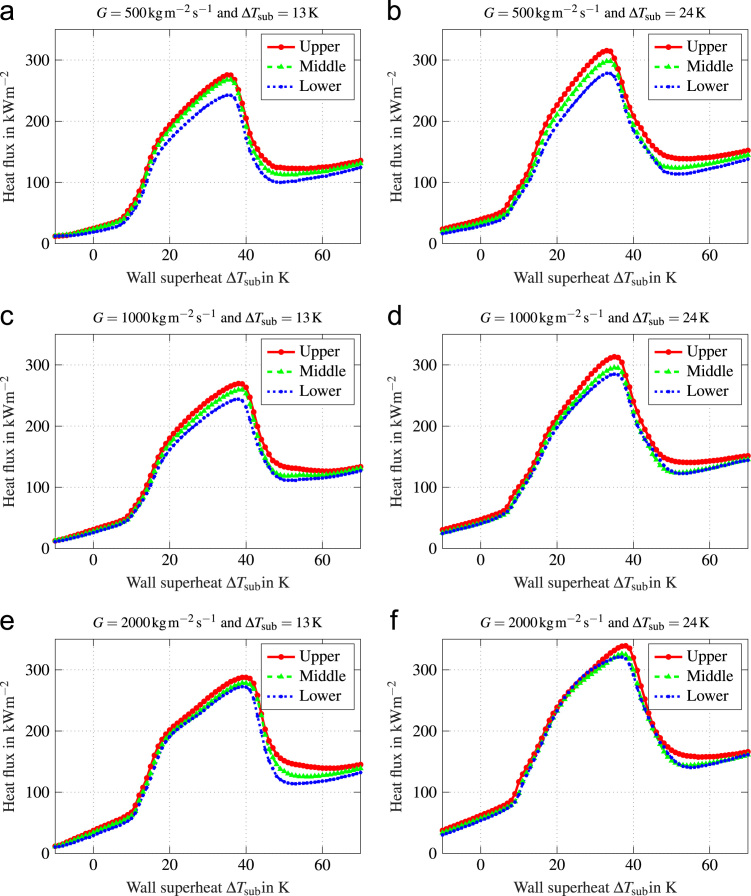
Average boiling curves for six combinations of operating parameters.

**Fig. 2:**
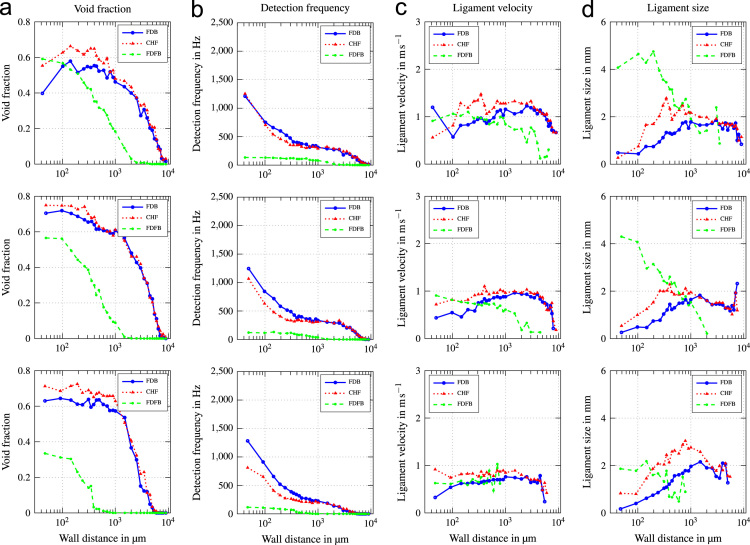
Void profiles at the upper, middle and lower measurement position (top to bottom) for $G=500\;\mathrm{kg}m^{-2}s{-}^{1}$ and $∆T_{\mathrm{sub}}=13\;K$.

**Fig. 3:**
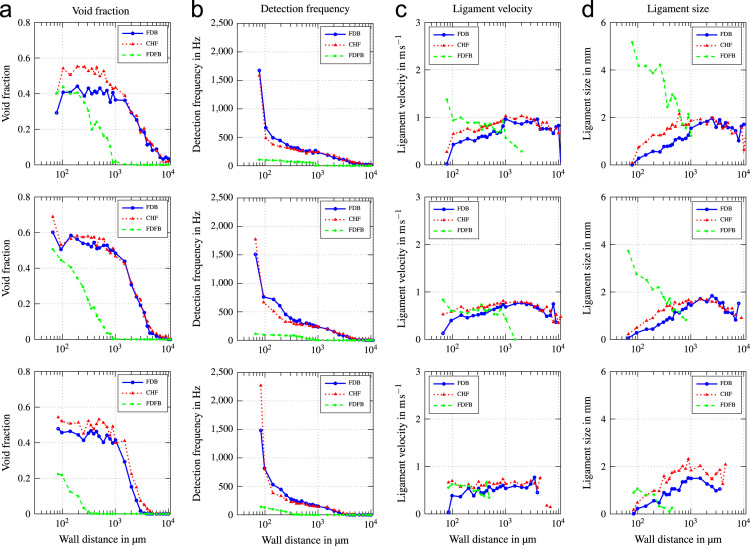
Void profiles at the upper, middle and lower measurement position (top to bottom) for $G=500\;\mathrm{kg}m^{-2}s^{-1}$ and $∆T_{\mathrm{sub}}=24\;K$.

**Fig. 4:**
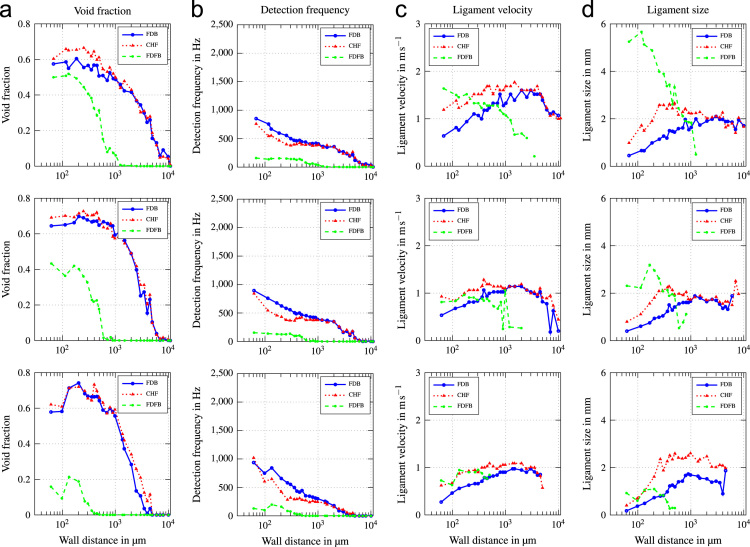
Void profiles at the upper, middle and lower measurement position (top to bottom) for $G=1000\;\mathrm{kg}m^{-2}s^{-1}$ and $∆T_{\mathrm{sub}}=13\;K$.

**Fig. 5:**
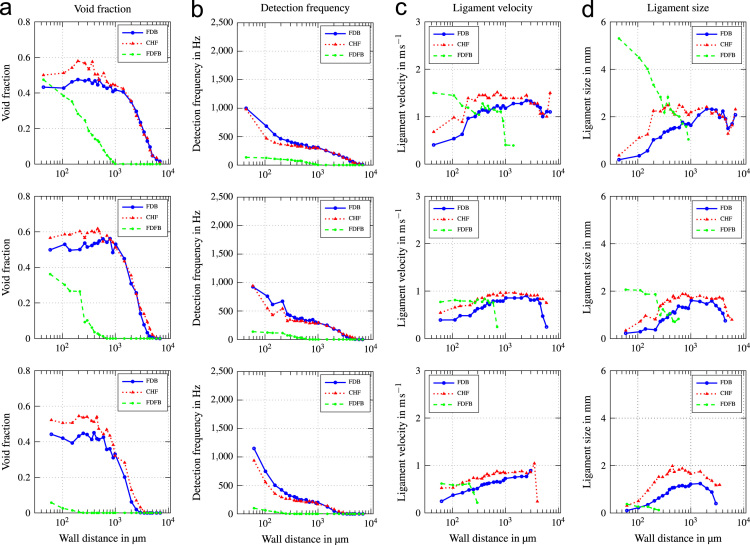
Void profiles at the upper, middle and lower measurement position (top to bottom) for $G=1000\;\mathrm{kg}m^{-2}s^{-1}$ and $∆T_{\mathrm{sub}}=24\;K$.

**Fig. 6:**
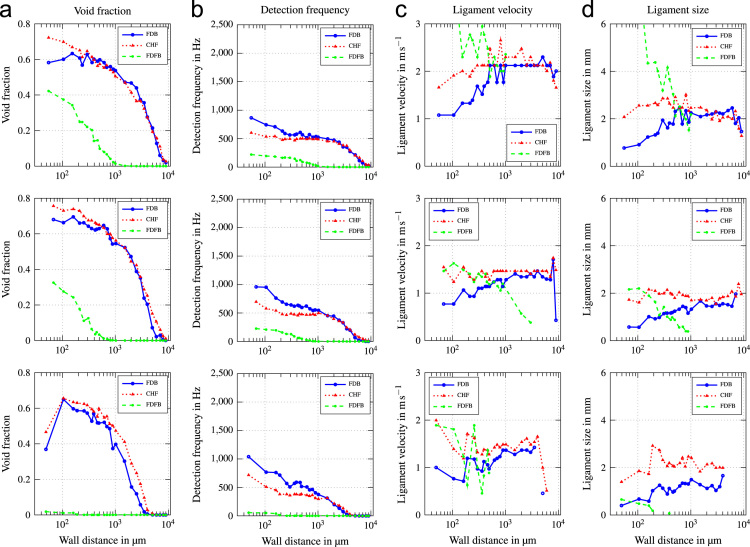
Void profiles at the upper, middle and lower measurement position (top to bottom) for $G\;=\;2000\;\mathrm{kg}m^{-2}s^{-1}$ and $∆T_{\mathrm{sub}}\;=\;13\;K$.

**Fig. 7:**
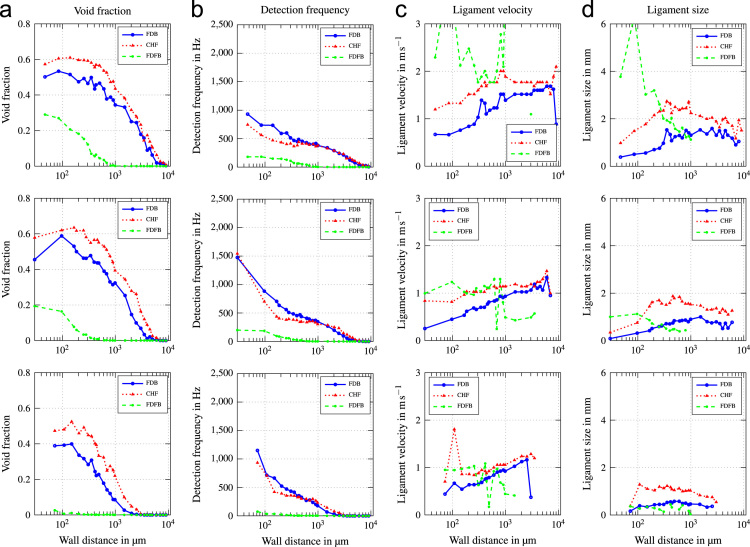
Void profiles at the upper, middle and lower measurement position (top to bottom) for $G=2000\;\mathrm{kg}m^{-2}s^{-1}$ and $∆T_{\mathrm{sub}}\;=\;24\;K$.

In this data article, average boiling curves and void profiles are presented. The data were obtained from experiments using a copper heater mounted flush in the wall of a vertical flow channel. Measurements were done at three different positions along the direction of flow. Experiments were conducted at mass fluxes of $G\;=\;500-2000\;\mathrm{kg}m^{-2}s^{-1}$, which corresponds to a superficial liquid flow velocity of $u_{l}=0.33-1.32\;ms^{-1}$. Inlet subcooling was varied between $∆T_{\mathrm{sub}}\;=\;13-24\;K$. Void profiles are provided for three characteristic states along the boiling curve, which are fully developed boiling (FDB), critical heat flux (CHF) and fully developed film boiling (FDFB). Fig. 1 shows average boiling curves for all operating conditions investigated. Fig. 2, Fig. 3, Fig. 4, Fig. 5, Fig. 6, Fig. 7 show void profiles at FDB, CHF and FDFB for each respective operating condition. The data presented in Fig. 1, Fig. 2, Fig. 3, Fig. 4, Fig. 5, Fig. 6, Fig. 7 are provided in the online version of this data article as text files with comma separated values. The files with the prefix “HeatFlux” contain heat flux data and are subsequently labeled according to “Prefix_MassFlux_Subcooling_MeasurementPosition.csv”. Row 1 of the files contains wall superheat in K and row 2 contains heat flux values in $\mathrm{kW}m^{-2}$. The files containing void profile data have a prefix named “VoidProfiles” and are labeled according to “Prefix_CharacteristicStateOnBoilingCurve_MassFlux_Subcooling_MeasurementPosition.csv”. Row 1 of these files contains the wall distance in μm, row 2 void fraction, row 3 detection frequency in hertz, row 4 ligament size in mm and row 5 ligament velocity in m/s.

### 2 Experimental design, materials, and methods

Experiments were conducted with a coolant at a heated copper wall in a square flow channel at mass fluxes of $G=500\;\;\mathrm{kg}m^{-2}s^{-1}$, $G=1000\;\mathrm{kg}m^{-2}s^{-1}$ and $G=2000\;\mathrm{kg}m^{-2}s^{-1}$ and an inlet subcoolings of $∆T_{\mathrm{sub}}=13\;K$ and $∆T_{\mathrm{sub}}=24\;K$. This section describes the experimental setup, the experimental procedure, the measurement techniques and the data analysis used.

#### 2.1 Boiling test rig

**Fig. 8:**
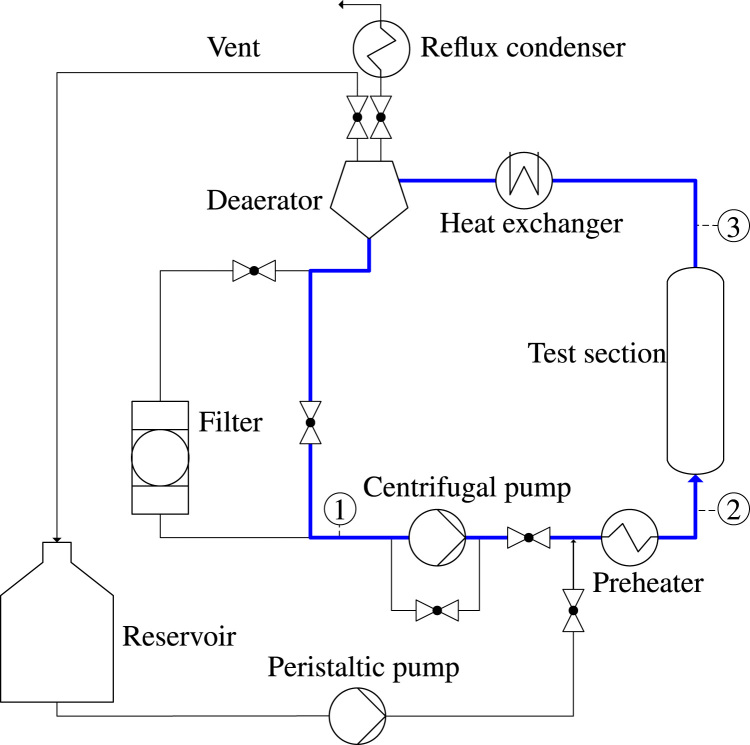
Schematic of the boiling loop (main flow loop is marked in bold blue).

Measurements were taken using a fluid loop with a vertically oriented test section, as shown schematically in Fig. 8. The fluid is circulated through the system by a centrifugal pump. Flow straighteners are placed below the test section to ensure homogeneous flow at the inlet. The absence of any secondary flows was confirmed via particle image velocimetry prior to conducting the experiments. A preheater controls the inlet subcooling of the fluid. Temperature measurements at position 1 and position 2 determine the amount of preheater power needed and measure the inlet subcooling of the fluid. After passing the test section, the two-phase flow enters a counter flow heat exchanger and is condensed and cooled down before entering a deaerator with a reflux condenser. The reflux condenser at the highest point of the test rig is kept open to environment. Thus, no pressure other than the hydrostatic pressure is imposed on the test section. Bypasses allow the filtering of particles immersed in the flow and enable the filling and the removal of fluid. Pressure transducers can be fitted at position 2 and position 3 to determine the inlet pressure of the test section and to test the tightness of the system to minimize the loss of the coolant during operation.

**Fig. 9:**
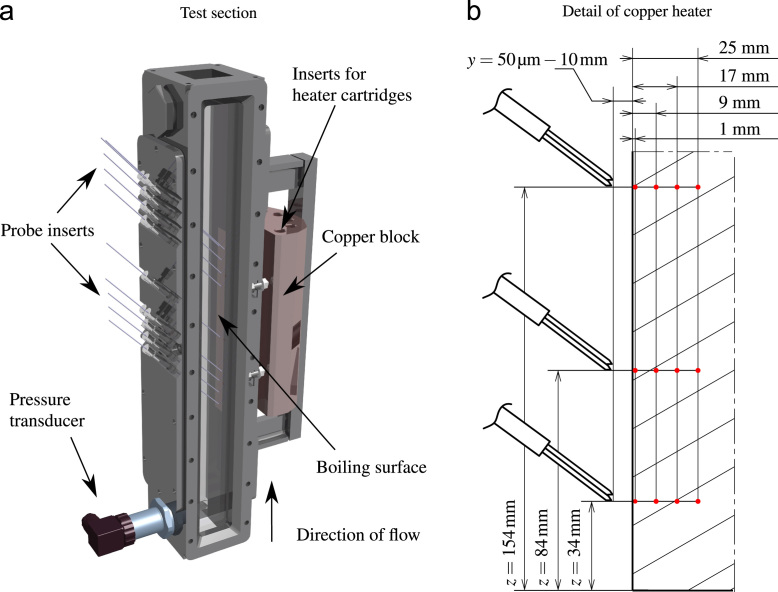
(a) Three-dimensional view of the test section, (b) detailed schematic view of the position of the fiber probes (double fiber probe configuration, not to scale) above the boiling surface and the thermocouples mounted inside the heater.

A three-dimensional view of the test section is shown in Fig. 9a. It has a total length in flow direction of 500mm and has a square cross section of 40mm by 40mm. Heat is provided by twelve heater cartridges with a combined maximum power of 2.4kW. They are inserted into the copper block on the right hand side of Fig. 9a. Heat is then transferred into the test section by a copper bar mounted flush in one of the walls of the test chamber. The boiling surface is placed in the axis of symmetry of the wall and has an area of 15mm by 200mm. The upstream end of the heater begins at 150mm after the inlet of the test section. The inlet pressure of the test section was measured to be 1.15bar. With the exception of the copper heater and glass inserts in the front and back of the test section, the test section is made of stainless steel. To be able to capture a full critical heat flux transient up to fully developed film boiling, a fluid with a low boiling temperature is used in the experiment. The fluid chosen is Novec 649 by 3 M, a dodecafluoro methylpentane similar in its chemical and physical properties to the common coolant FC-72. It has a boiling point of 49°C and an enthalpy of evaporation of $88{\;\mathrm{kJkg}}^{-1}$ at 1.0bar ambient pressure. Its main physical properties in comparison to water are given in Table 1 for saturation conditions at the inlet pressure of 1.15bar.

**Table 1:** Main fluid parameters at saturation conditions for 1.15 bar of Novec 649 in comparison to water.

Quantity	Novec 649	Water	Unit
$T_{\mathrm{sat}}$	52.7	103.6	°C
${∆h}_{l,g}$	86.9	2247.1	${\mathrm{kJ kg}}^{-1}$
$c_{p,l}$	1121.45	4221.28	${\mathrm{J kg}}^{-1}K^{-1}$
$c_{p,g}$	908.9	2092.1	${{\mathrm{J kg}}^{-1}K}^{-1}$
$\rho_{l}$	1504.78	955.77	$\mathrm{kg}m^{-3}$
$\rho_{g}$	12.21	0.67	$\mathrm{kg}m^{-3}$
σ	10.8	72.8	$\mathrm{mN}m^{-1}$
$T_{\mathrm{crit}}$	441.81	647.1	K
$p_{\mathrm{crit}}$	1.88	22.12	MPa

#### 2.2 Measurement techniques

##### 2.2.1 Temperature and heat flux

To measure heat flux and wall temperature, there are three rows of thermocouples inside the copper bar. Each row consists of four thermocouples placed at different distances below the surface according to Fig. 9b. The thermocouples are mounted in the heater׳s axis of symmetry. Surface temperature is extrapolated quadratically using the temperature gradient between the thermocouples in each respective row. A numerical analysis of the heat transfer processes inside the heater during operation showed no substantial heat transfer inside the heater in the direction of flow. Heat flux is therefore calculated for each row using an inverse heat transfer algorithm based on the law of Fourier factoring in the transient heating of the copper bar in wall normal direction according to Eq. (1). Fig. 9b shows a detailed view of the thermocouple positions within the heater. Thermocouples of type K are used at distances of y=9mm, y=17mm and y=25mm below the heater surface, while the ones closest to the boiling surface at a distance of y=1mm are of type T. The acquisition frequency for all temperature measurements in the system is 2Hz.

(1) $$\dot{q}\;=\;-\lambda_{\mathrm{cu}}\frac{\Delta T}{\Delta y}-\rho_{\mathrm{cu}}{\Delta y}_{\mathrm{cu}}\frac{\Delta T}{\Delta t}$$

##### 2.2.2 Optical fiber probes

**Fig. 10:**
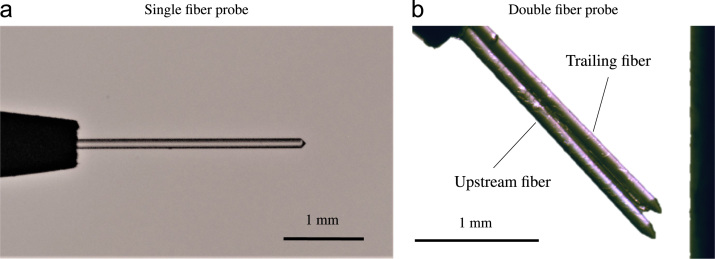
Microscopic images of the two types of optical fiber probes used in this study.

Three optical fiber probes were used simultaneously to measure void data at distances between y≈50µm and y≈10mm normal to the wall. Based on the law of Snellius [1], these probes detect the change in refractive index at the tip of the glass fiber. When a bubble touches the tip of the probe, light propagating within the fiber is reflected. Correspondingly, when liquid is present at the tip, the light is projected into the flow channel. This results in a series of light pulses for intermittent two-phase flow at the tip, which can then be analyzed. Optical fiber probes have been used successfully in many two-phase flow experiments [2], [3], [4], [5], [6], [7], [8]. Fig. 10a and b show the two types of probes used in this study. The single fiber probe can be used to measure void fraction and ligament frequency, whereas the double fiber probe provides additional quantitative data on void ligament velocity and size. The probe tips consist of either one or two glass fibers with a core diameter of y=50µm per fiber. Each fiber has a conical shape at an angle of approximately 45°. The fibers are embedded in a steel ferrule for increased mechanical stability. For the double fiber probes, the two individual glass fibers are glued together to ensure a fixed distance between the tips of each fiber. For both configurations, the measurement apparatus consists of the probe tip, a fiber coupled laser source, a fiber coupler, a photodiode and an amplifier as well as an analog to digital converter running at 60kHz for each fiber. A fully fiber-based beam path ensures a good signal to noise ratio and minimizes unwanted interference.

The optical fiber probes were inserted into the flow channel at an angle of 42° from the left-hand side of the test cell as depicted in Fig. 9a. The probe tips were positioned at the heights of the thermocouple rows as shown in Fig. 9b. This allowed for the locally coupled synchronous measurement of heat flux, temperature data and void data. A traversing mechanism with an accuracy of ±5µm was used to position the probes at variable distances from the heater surface. The position of each probe was optically verified with microscopic images for each measurement point. Due to the high thermal conductivity of the copper heater, boiling for each measurement started at the same position at the very upstream end of the heater. No variance of the point of the onset of boiling was observed. Therefore, the fiber probes always captured the boiling process at the same stage along the direction of flow.

**Fig. 11:**
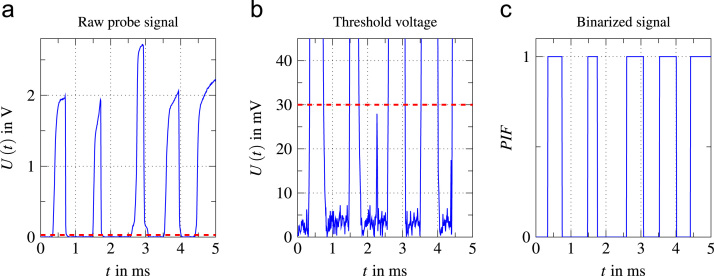
Binarization sequence from raw signal to the binary signal using a threshold voltage (red dashed line).

All void data are calculated based on a phase indicator function (PIF) according to Eq. (2). Fig. 11 shows an exemplary binarization sequence using Eq. (2). Based on the raw signal from bubbles touching the probe tip, a threshold voltage $U_{\mathrm{th}}$ is set above the noise floor in the raw signal. Using this voltage, the raw signal is binarized. A value of PIF=0 corresponds to the liquid phase and a value of PIF=1 to the void phase. This approach has been used similarly in other works, for example [9], [10], [11] as well as for the well-known experimental database by Garnier et al. [12]. A moving average approach was used to calculate mean values for a given time interval $∆t_{i}$ containing a number of samples n. For the purpose of this study, n=30,000 was used, which for a sample rate of sr=60kHz equates to an averaging interval of $∆t_{i}=0.5\;s$. This interval length was chosen to match the acquisition frequency of 2Hz for temperature data. The calculation of the void data in each interval was done as follows.

(2) $$\mathrm{PIF}\;=\;\{\begin{array}{c} 1\;\forall \;U>U_{\mathrm{th}}\\ 0\;\forall \;U\leq U_{\mathrm{th}} \end{array}$$

##### 2.2.3 Void fraction

The local void fraction α was calculated according to Eq. (3) for any time interval $∆t_{i}$ by firstly evaluating Eq. (2) for each recorded sample n. The ratio of the sum over each PIF(j) to the total number of recorded samples n in the time interval is then equal to α during $∆t_{i}$. This approach has been used before by other authors for the calculation of the void fraction from fiber probes, for example [13], [14].

(3) $$\alpha \left(\Delta t_{i}\right)=\frac{1}{n}\overset{n}{\underset{j=1}{\sum }}\mathrm{PIF}\left(\left(j\right)\right)$$

##### 2.2.4 Detection frequency

The detection frequency $f_{\mathrm{lig}}$ in this work is defined as the total number of detected void ligaments $N_{\mathrm{lig}}$ per time interval $∆t_{i}$ according to Eq. (4).

(4) $$f_{\mathrm{lig}}\left(\Delta t_{i}\right)=N_{\mathrm{lig}}/\Delta t_{i}$$

##### 2.2.5 Contact time

**Fig. 12:**
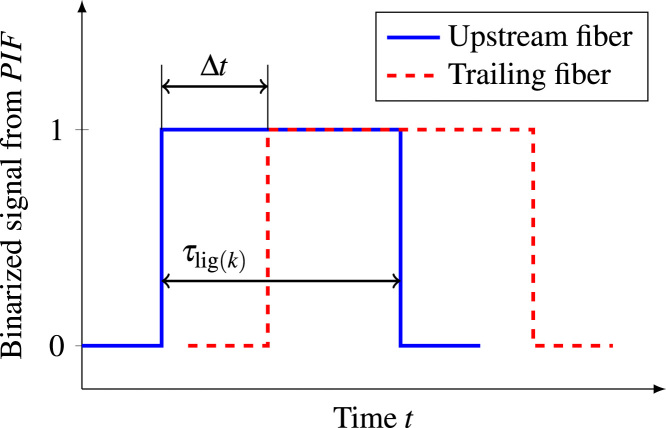
Schematic depiction of the time difference between the signals from the upstream and trailing probe used for the calculation of void velocity.

For the calculation of void ligament velocity and size, the average contact time $\tau_{\mathrm{lig}}$ of ligaments per time interval is needed. The contact time for a single ligament k is calculated by Eq. (5), where $n_{\mathrm{lig}\left(k\right)}$ is the number of samples with a value of PIF=1 for each ligament k. This is schematically depicted in Fig. 12.

(5) $$\tau_{\mathrm{lig}(k)}\;=\;n_{\mathrm{lig}(k)}/\mathrm{sr}$$

The average contact time for each time interval is then calculated as:

(6) $$\tau_{\mathrm{lig}}\left(\Delta t_{i}\right)=\frac{1}{N_{\mathrm{lig}}}\overset{N_{\mathrm{lig}}}{\underset{k=1}{\sum }}\tau_{\mathrm{lig}(k)}$$

##### 2.2.6 Ligament velocity

The average ligament velocity $u_{\mathrm{lig}}$ is calculated using a time-of-flight method, similar to the approach used in [12]. The time ∆t it takes a void interface to cover the distance $l_{\mathrm{probe}}$ between the upstream fiber and trailing fiber can be extracted from the data from the double fiber probe setup. This is schematically shown for a generic binarized double fiber probe signal in Fig. 12. By cross correlating the signals of the two fibers for a given time interval, the mean time of flight $\overset{®}{∆t}$ for all void ligaments in this interval is calculated. The vertical distance between the two fibers for all double fiber probes used in this study was in the range of $l_{\mathrm{probe}}=200-220\;\mathrm{µm}$. This value is a compromise between the miniaturization of the probe and the distance needed to accurately measure the time of flight of the interface. The average ligament velocity of a given time interval is then calculated as:

(7) $$u_{\mathrm{lig}}\left(\Delta t_{i}\right)=\;l_{\mathrm{probe}}/\bar{\Delta t_{i}}$$

##### 2.2.7 Ligament size

Assuming spherical shape, the mean ligament size $l_{\mathrm{lig}}$ for each interval is calculated by multiplying the mean velocity $u_{\mathrm{lig}}$ with the mean contact time $\tau_{\mathrm{lig}}$.

(8) $$l_{\mathrm{lig}}(\Delta t_{i})\;=\;\tau_{\mathrm{lig}}\cdot u_{\mathrm{lig}}$$

#### 2.3 Uncertainty

**Fig. 13:**
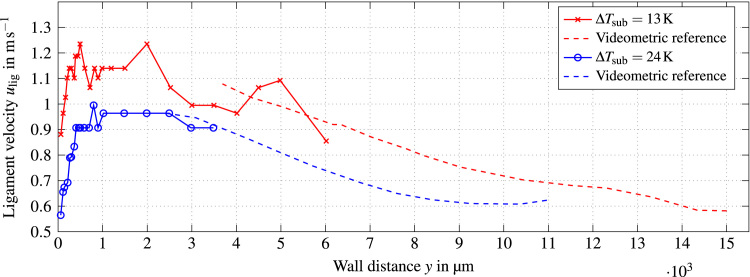
Comparison between the void velocity obtained through videometric reference experiments and the data from double fiber probes for two subcoolings. Mass flux was kept constant at $G=1000\;\mathrm{kg}m^{-2}s^{-1}$.

Calibration experiments were conducted prior to this study. A deviation of approximately 0.2K between any thermocouples used in the test rig at ambient conditions was measured. The position accuracy of the thermocouples inside the heater due to manufacturing inaccuracies was assumed to be better than 0.1mm. A Gaussian error propagation was calculated based on these values and yielded absolute uncertainties of $\pm 10.7\;\mathrm{kW}m^{-2}$ for surface heat flux and ±0.9K for wall temperature. A statistical fluctuation of $20\;\mathrm{kg}m^{-2}s^{-1}$ for the mass flux was tolerated as a negligible influence on CHF and wall temperature was observed in reference experiments. Videometric calibration experiments during the development of the fiber probe tips showed good agreement between the probe signals and the observed trajectories of bubbles. Typical errors commonly found in literature (e.g. [3], [8], [15]) were matched regarding quantities derived from Eq. (2). The velocity measurements from the double fiber probes were compared to bubble velocities obtained by stereoscopic videometry for two-phase flow with single bubbles. A deviation of less than ±15% was observed between the two measurement techniques. However, the only well-defined parameter in this context is the interface velocity of single bubbles. This quantity is not directly related to the actual void velocity [16]. As described by Garnier et al. [12], the true velocity of the void phase can only be related to the distance between the two fibers of a double fiber probe if the velocity and size distributions are spatially uniform where the void is intercepted by the two fibers. The mean void velocity can then be derived from a double fiber probe setup when being calculated over a sufficiently large number of bubbles and assuming that void velocity does not depend on ligament diameter. This was verified in videometric reference experiments. In these experiments, mass flux was kept constant at $1000\;\mathrm{kg}m^{-2}s^{-1}$ and two different subcoolings were investigated. Velocity profiles from the double fiber probe setup were compared to high-speed videometry measurements. The data from double fiber probes was evaluated for a time interval of 0.5s to match the acquisition frequency of temperature data of 2Hz. The number of detected void ligaments during this period was in the order of $N_{\mathrm{lig}}=200-500$. The bulk flow velocity for these conditions is approximately $0.66\;ms^{-1}$. Videometric measurements near the heater surface were not possible due to high vapor concentration. Measurements with the double fiber probes were inhibited in regions of low gas holdup due to the subsequent small number of detected bubbles. The results are shown in Fig. 13. Both measurement techniques show good agreement in the region where they overlap with a maximum deviation of approximately ±10%. The more conservative uncertainty from the single-bubble experiments of 15% for the average ligament velocity is chosen for this study. The uncertainties of quantities presented in this paper are summarized in Table 2.

**Table 2:** Overview of uncertainties of measured quantities.

Quantity	Uncertainty	Unit
Void data from PIF	±2.5	%
Ligament velocity	±15.0	%
Position of void probes	±5	μm
Heat flux	±10.7	$\mathrm{kW}m^{-2}$
Wall temperature	±0.9	K
Mass flux	±20	$\mathrm{kg}m^{-2}s^{-1}$
Subcooling	±0.2	K

#### 2.4 Measurement procedure

Experiments were conducted transiently. Preliminary tests showed that void data from transient runs closely matches steady-state experiments for the fully developed boiling regime. This confirmed the quasi-static behavior reported in earlier studies with a previous development stage of the current experimental setup in [17]. To ensure consistent surface properties, the boiling surface was polished after each fourth experiment using a 2500-grit paper. The first run after polishing was discarded to eliminate the effects of non-condensables. This way, a very good repeatability of the experimental data was achieved. The experimental procedure for each experiment was as follows:

1. The heater is at or close to ambient conditions. Fluid is circulated through the system. No boiling activity is present on the heater surface.
2. The system is heated up to the chosen subcooling.
3. After reaching the set subcooling, the fiber probes are positioned at target distances of 50µm to 50mm above the boiling surface at the positions of the thermocouple rows. The positioning accuracy of the traversing mechanism is verified optically before each experiment.
4. The transformer powering the heater cartridges is switched on simultaneously with the start of data acquisition. The system goes through one complete boiling cycle from single-phase convection to fully developed film boiling. The transformer is switched off when the Leidenfrost point is reached and surface heat flux begins to increase again. Data acquisition is stopped.
5. The system is cooled down until the initial conditions are reached.

#### 2.5 Definition of points along the boiling curve

The data are provided for three generic points along the boiling curve, the point of fully developed boiling (FDB), at CHF and at fully developed film boiling (FDFB). The respective definitions as a function of wall superheat $∆T_{w}=T_{w}-T_{\mathrm{sat}}$ are given in Table 3. CHF in this work is defined as the maximum surface heat flux observed in the experiments, corresponding to other works, for example [14]. For each transient experiment, the point of maximum heat flux for each thermocouple row is calculated from the experimental data. Heat flux, wall temperature and void data at CHF are extracted using the obtained time stamp. Values at FDB and FDFB are then calculated using the respective wall temperature at CHF.

**Table 3:** Definition of points along the boiling curve for which void data profiles are presented.

Point	Definition
FDB	$∆T_{w,\mathrm{FDB}}\;=\;75\%∆T_{w,\mathrm{CHF}}$
CHF	$∆T_{w,\mathrm{CHF}}\;=\;∆T_{w}\mathrm{at}\dot{q}\;=\;{\dot{q}}_{\max}$
FDFB	$\dot{q}\;=\;{\dot{q}}_{\min}\mathrm{at}∆T_{w,\mathrm{FDFB}}>∆T_{w,\mathrm{CHF}}$
